# Kaposi's Sarcoma Herpesvirus Upregulates Aurora A Expression to Promote p53 Phosphorylation and Ubiquitylation

**DOI:** 10.1371/journal.ppat.1002566

**Published:** 2012-03-01

**Authors:** Qiliang Cai, Bingyi Xiao, Huaxin Si, Amanda Cervini, Jianming Gao, Jie Lu, Santosh K. Upadhyay, Suhbash C. Verma, Erle S. Robertson

**Affiliations:** 1 Department of Microbiology and the Tumor Virology Program, Abramson Comprehensive Cancer Center, Perelman School of Medicine at the University of Pennsylvania, Philadelphia, Pennsylvania, United States of America; 2 Department of Microbiology and Immunology, School of Medicine, University of Nevada, Reno, Nevada, United States of America; University of Texas Health Science Center San Antonio, United States of America

## Abstract

Aberrant expression of Aurora A kinase has been frequently implicated in many cancers and contributes to chromosome instability and phosphorylation-mediated ubiquitylation and degradation of p53 for tumorigenesis. Previous studies showed that p53 is degraded by Kaposi's sarcoma herpesvirus (KSHV) encoded latency-associated nuclear antigen (LANA) through its SOCS-box (suppressor of cytokine signaling, LANA^SOCS^) motif-mediated recruitment of the EC_5_S ubiquitin complex. Here we demonstrate that Aurora A transcriptional expression is upregulated by LANA and markedly elevated in both Kaposi's sarcoma tissue and human primary cells infected with KSHV. Moreover, reintroduction of Aurora A dramatically enhances the binding affinity of p53 with LANA and LANA^SOCS^-mediated ubiquitylation of p53 which requires phosphorylation on Ser215 and Ser315. Small hairpin RNA or a dominant negative mutant of Aurora A kinase efficiently disrupts LANA-induced p53 ubiquitylation and degradation, and leads to induction of p53 transcriptional and apoptotic activities. These studies provide new insights into the mechanisms by which LANA can upregulate expression of a cellular oncogene and simultaneously destabilize the activities of the p53 tumor suppressor in KSHV-associated human cancers.

## Author Summary

Aurora kinases are evolutionally conserved serine/theronine kinases that regulate cell mitotic progression in eukaryotic cells. Aurora kinase A, B and C were identified in mammalian cells. Among them, Aurora A was first known to regulate genomic instability and tumorigenesis, and is frequently amplified in multiple human cancers. Aurora-kinase inhibition has been shown to effectively block cell growth and induce death of cancer cells. Kaposi's sarcoma-associated herpesvirus (KSHV) encoded latency-associated nuclear antigen (LANA) is essential for KSHV-induced transformation of primary human B-lymphocytes and endothelial cells. In this study, we discovered that LANA remarkably enhances Aurora A production, and that elevated Aurora A acts as a negative regulator to induce phosphorylation and LANA-mediated ubiquitylation of p53. Importantly, inhibition of Aurora A production leads to cell death of KSHV-positive B lymphoma cells. This study clearly demonstrates that Aurora A is targeted by an oncogenic virus for inhibition of p53 function, and is a potential target for viral associated cancer therapy.

## Introduction

Kaposi's sarcoma-associated herpesvirus (KSHV), also named human herpesvirus 8, is a member of the gamma-herpesviruses and is associated with Kaposi's sarcoma (KS), multicentric Castleman's disease (MCD) and primary effusion lymphoma (PEL) [1]–[4]. Studies have shown that PELs are dependent on KSHV for survival, as loss of the KSHV genome results in cell death [5]. These findings demonstrate that KSHV infection can reprogram cellular gene function and thereby mediate viral oncogenesis. KSHV is predominantly latent in most cells in KSHV-associated lesions and during latency only a few viral genes are expressed. The latency associated nuclear antigen (LANA) encoded by open reading frame (ORF) 73, is one of the critical KSHV encoded latent antigens, and is expressed in viral infected tumor cells of KSHV-associated malignancies [6], [7]. LANA plays a multifunctional role contributing to viral persistence and tumorigenesis through targeting DNA replication, chromosome tethering, anti-apoptosis, cell cycle regulatory, and gene regulatory functions [8]–[13]. At the gene transcription level, LANA exerts broad repressive or activation effects by interacting with a number of transcriptional factors including mSin3A, CBP, RING3, GSK-3β and p53 for its transcription repression activities [8], [14]–[16], and E2F, Sp1, RBP-Jκ, ATF4, CBP, Id-1, and Ets to drive transcriptional activation [17]–[22].

Aurora A, a centrosome-associated Serine/Threonine oncogenic kinase, was first identified as a human homologue of the Aurora/Ipl1p kinase family [23]. The human Aurora A gene is located at chromosomal region 20q13.2 and contains a 1209-bp open reading frame that encodes 403 amino acids with a molecular weight of 46 kDa [24]. The promoter of Aurora A contains three putative binding sites for transcription factors: E2F, Sp1 and Ets [25]. Aurora A localizes around centrosomes during interphase and prophase, on the microtubules near spindle poles in metaphase and the polar microtubles during anaphase & telophase [26]. Aurora A participates in multiple functions associated with mitotic events, including centrosome maturation and separation, bipolar spindle assembly, chromosome alignment and cytokinesis [27]. Enhanced expression of Aurora A can lead to centrosome amplification and aneuploidy as a results of incomplete cytokinesis, which results in either cell death or survival through malignant transformation in a p53-dependent manner [28], [29]. Aberrant expression of Aurora A has been reported in a wide variety of tumor types and in most human cancer cell lines [24], [28], [30]–[32]. A number of substrates of Aurora kinase A have been identified, such as TPX2 [33], Eg5 [34], CDC25B [35], p53 [36] and BRCA-1 [37].

Like the aberrant expression of Aurora A, loss of p53 function also induces similar phenotypes of centrosome amplification and aneuploidy in cells [38], [39]. It is well known that wild type p53 is able to induce growth arrest or apoptosis when cells are exposed to stress, and p53 is frequently mutated or deleted in human cancers [38], [39]. As a substrate of Aurora A kinase, p53 can be phosphorylated on both Ser215 and Ser315 and this leads to destabilization and inhibition of p53 via the Mdm2-mediated ubiquitylation and proteasomal degradation pathway [36], [40]. In KSHV latently infected cells, our previous studies have shown that p53 can be degraded by the cellular EC_5_S ubiquitin complex-mediated pathway targeted by the SOCS motif of LANA [41]. In this study, we further show that the protein levels of Aurora A kinase is upregulated by LANA, and that elevated Aurora A induces phosphorylation of p53 which enhances the interaction of LANA with p53, and promotes LANA-mediated p53 ubiquitylation and degradation, and hence inhibition of p53 transcriptional and apoptotic activities.

## Results

### Aurora A expression is upregulated in the LANA-expressing cells

**Figure 1:**
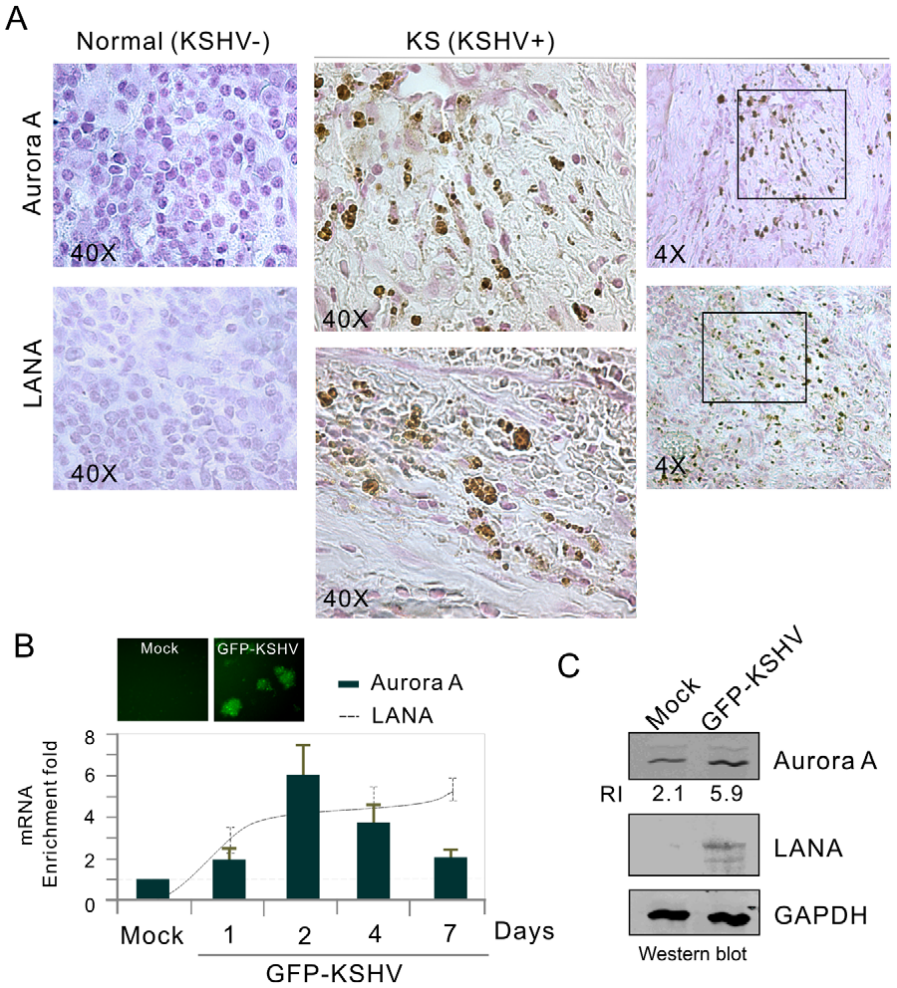
Aurora A expression is up-regulated in KSHV latently infected cells. (**A**) The protein levels of Aurora A were enhanced in KSHV-associated KS patient skin tissue. Kaposi's sarcoma tissue specimens from 3 patients were analyzed for both LANA and Aurora A expression by immunohistochemistry. A representative sample of Kaposi's sarcoma patient (middle and right panels) and the adjacent normal tissue (left panel) are shown. (**B**) Quantitative real-time PCR analysis of Aurora A and LANA transcripts in PBMC with GFP-KSHV infection. Total RNA was isolated from cells with GFP-KSHV infection for 0, 1, 2, 4 and 7 days. Real-time PCR was performed as described in Materials and Methods. The relative levels of Aurora A and LANA transcript were calculated by the cycle threshold (ΔΔCt) values and shown by the fold change compared to mock (day 0). All samples were tested in triplicate and the calculation of the mean and standard deviation from two separate experiments. The mock cells without or with GFP-KSHV infection for 4 days were shown on the top. (**C**) Immunoblotting analysis. Whole cell lysate of human PBMC cells with GFP-KSHV infection for 4 days or mock, were subjected to immunoblotting with antibodies against LANA, Aurora A and GAPDH. The relative instensity (RI) of Aurora A is quantified and shown in the figure.

**Figure 2:**
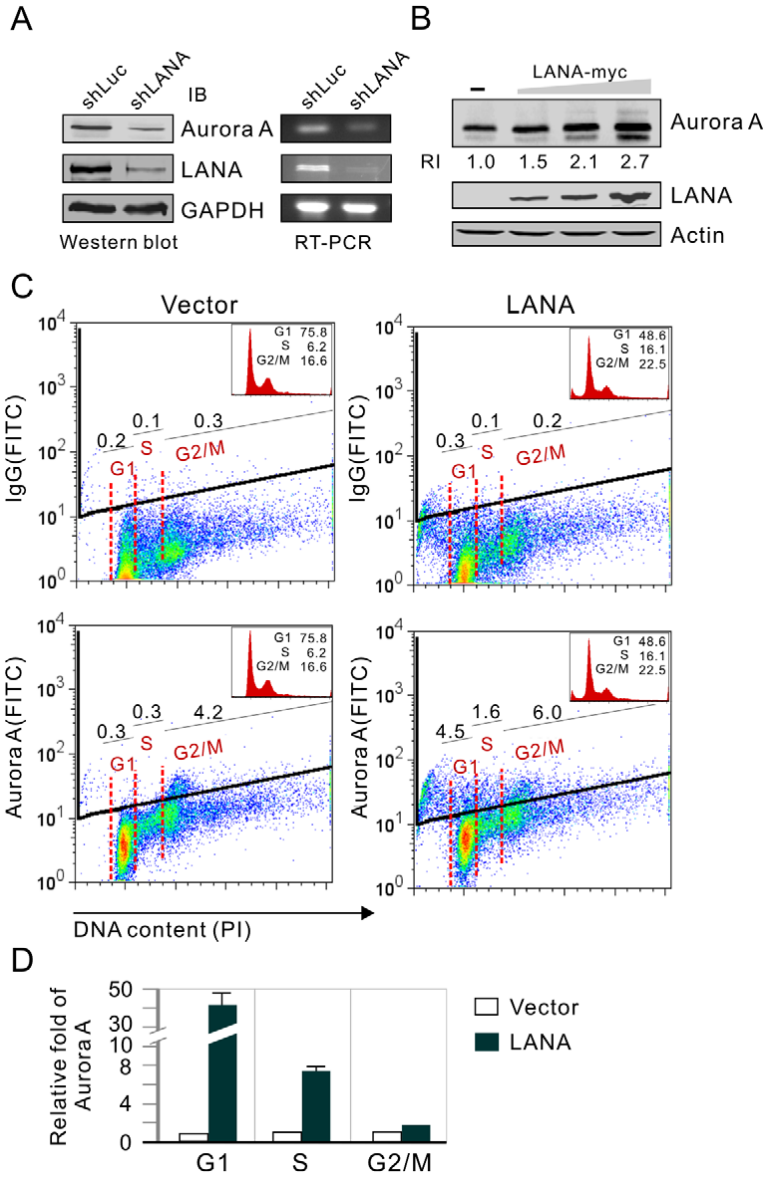
Aurora A transcript is dramatically enhanced in a LANA-dependent manner. (**A**) The level of Aurora A expression was decreased in BC3 cells with LANA knockdown. Cell lysates from BC3 cells with LANA (shLANA) or control firefly luciferase knockdown (shLuc) were subjected to immunoblotting against LANA, Aurora A and GAPDH, or total RNA were extracted for RT-PCR analysis. (**B**) LANA increases endogenous Aurora A expression in a dose-dependent manner. Fifteen million 293 cells were transiently transfected either with empty vector or increasing amounts of pA3M-LANA. At 36 hours post-transfection, the cells were harvested, lysed and individually subjected to western blot with antibodies against Aurora A, LANA and Actin. (**C**) Cytometric profile of Aurora A expression in HeLa cells in the presence or absence of LANA. HeLa cells transfected with pA3F-LANA or pA3F vector alone were individually divided and subjected to propidium iodide (PI)-staining cell cycle analysis (inset) combined with anti-Aurora A (FITC) or normal IgG (FITC) staining. A representative of double staining with the gating percentage of Aurora (IgG) expression at G1, S or G2/M phase of cell cycle was shown. (**D**) The relative fold of Aurora expression in each phase of HeLa cells with LANA expression or vector alone from panel C was presented and normalized by IgG staining control. Data from two repeat experiments.

Aurora A has been shown to aberrantly accumulate and inhibit p53 function in most cancer cells. To analyze whether LANA-mediated inhibition of p53 in KSHV latently infected cells is associated with Aurora A, we first tested the protein levels of Aurora A in KSHV positive KS tumor samples and KSHV negative normal tissues by immunohistochemistry assays. The results showed that Aurora A expression is highly expressed in KS patient tissue and not in normal tissue (Figure 1A). The observation from de novo infection of human primary cells with KSHV *in vitro* shows that although there was an overall increase of Aurora A mRNA transcript and protein levels up to 7-days post-infection, the peak level of Aurora A was at 2-days post-infection (Figure 1B and C). This strongly indicates that elevated expression of Aurora A was indeed associated with KSHV infection, and that the early rapid enhancement of Aurora A expression could be important for KSHV to establish long term latent infection. To elucidate the role of LANA on Aurora A expression, LANA in KSHV-positive BC3 cell line was transiently knocked down by introduction of small interference RNA specifically against LANA without interrupting other latent transcripts (supplementary Figure S1). The levels were monitored by western blot analysis. The results showed that the protein levels of Aurora A were greatly decreased once LANA expression was reduced (Figure 2A, left panel). In addition, a significant reduction in Aurora A transcripts was observed after LANA was inhibited (Figure 2A, right panel), supporting the hypothesis that LANA can modulate the transcriptional activity of Aurora A promoter and so regulate Aurora A expression. This is further confirmed by the fact that LANA induces a dose-dependent increase in Aurora expression in HEK293 cells with transient transfection (Figure 2B). To determine which phase of cell cycle expressing Aurora A is affected by LANA, the levels of Aurora A at different cell cycle phases in the presence or absence of LANA were determined by flow cytometric analysis. The results showed that LANA dramatically enhanced Aurora A expression at G1 and S phases but mildly so at G2/M phase (Figure 2C and D). This indicated that LANA can play a role in maintaining higher level of Aurora A at different phases of cell cycle.

### LANA upregulates Aurora A transcripts by targeting the Sp1 *cis* element within the promoter

Given the modulation of Aurora A expression by LANA, we constructed a luciferase reporter driven by Aurora A promoter (position −527 to +387) (supplementary Figure S2A), and performed reporter assays in the presence or absence of LANA in both HEK293 and DG75 cells. The results showed that LANA dramatically enhanced the transcription level of the Aurora A promoter when compared to vector control (supplementary Figure S2B), which was further corroborated by a dose-dependent induction (supplementary Figure S2C).

**Figure 3:**
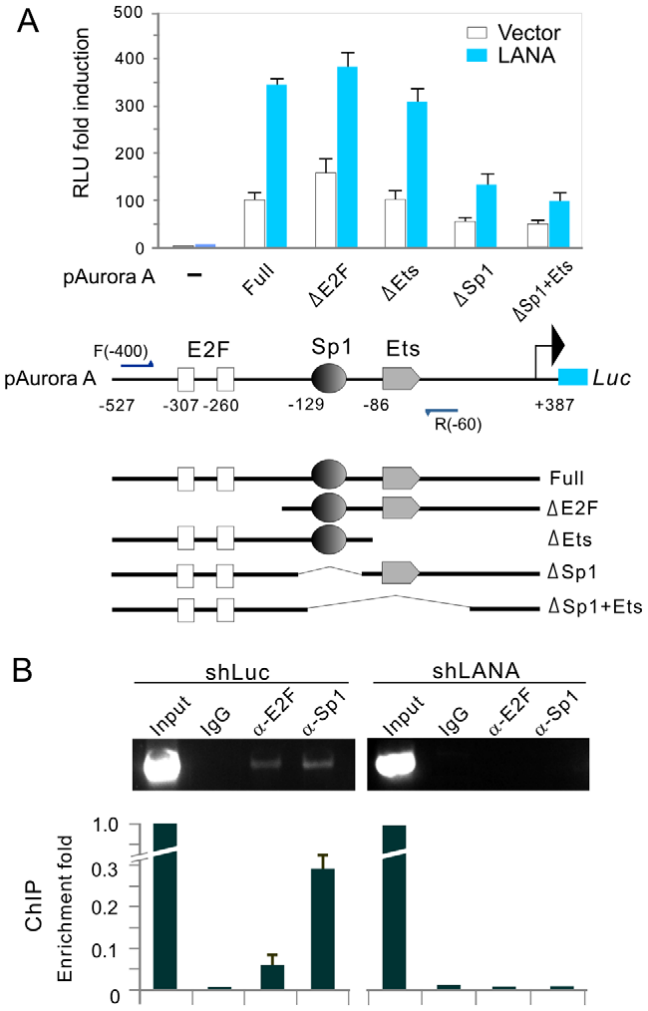
The Sp1 *cis*-acting element is critical for LANA-mediated enhancement of Aurora A transcription. (**A**) HEK293 cells were co-transfected full length Aurora A promoter or its mutants driving reporter plasmid with either pA3M-LANA or pA3M vector. At 24-hrs post-transfection, cells were harvested and subjected to reporter assay. The schematic representation of full length Aurora A gene promoter or its mutants-driven luciferase constructs is shown at the bottom panel. The results were presented by the RLU (relative luciferase unit) fold compared to pGL3-basic with vector alone. Data is presented as means±SD of three independent experiments. (**B**) ChIP analysis of endogenous Aurora A promoter in BC3 cells with or without LANA knockdown. ChIP analysis was conducted using normal mouse IgG, α-E2F, α-Sp1 agarose, and subjected to PCR analysis using primer F(−400) and R(−60) indicated in A. The results of quantitative real-time PCR are shown at the bottom panel.

A transcription factor analysis and previous studies identified three *cis* elements E2F, Sp1 and Ets within the Aurora A promoter [42]. To define which *cis* element is critical for LANA to activate Aurora A transcription, a series of mutants of the Aurora A promoter driving the luciferase reporter gene were generated and subjected to reporter assays in HEK 293 cells in the presence or absence of the LANA. As shown in Figure 3A, in the presence of LANA, the Aurora A promoter with specific deletions of E2F or Ets transcription factor-related locus (ΔE2F or ΔEts) resulted in a mild difference in stimulation of Aurora A promoter activity when compared to the wild type Aurora A promoter (E2F+Sp1+Ets). However, the Aurora A promoter with deletion of both Sp1 and Ets elements (ΔSp1+Ets) or Sp1 alone (ΔSp1) led to a remarkable decrease in the promoter activity (Figure 3A). These results strongly suggested that the Sp1 responsive element within the Aurora A promoter is a major cis element modulated by LANA. To further confirm that the Sp1-binding site within the Aurora A promoter is a major target for LANA, we performed chromatin-immunoprecipitation assays with Sp1 or E2F specific antibodies using BC3 cells with or without LANA knockdown. Consistently, the results showed that Sp1 had a much higher affinity than E2F when compared to the non-specific IgG control in binding to Aurora A promoter (Figure 3B). Inhibition of LANA expression dramatically reduced the association of Sp1 to its cis-acting element (Figure 3B). This suggests that LANA can enhance Aurora A transcript levels through targeting of the Sp1 cis-element within the promoter.

### Aurora A is important for LANA to inhibit p53 transcriptional activity

**Figure 4:**
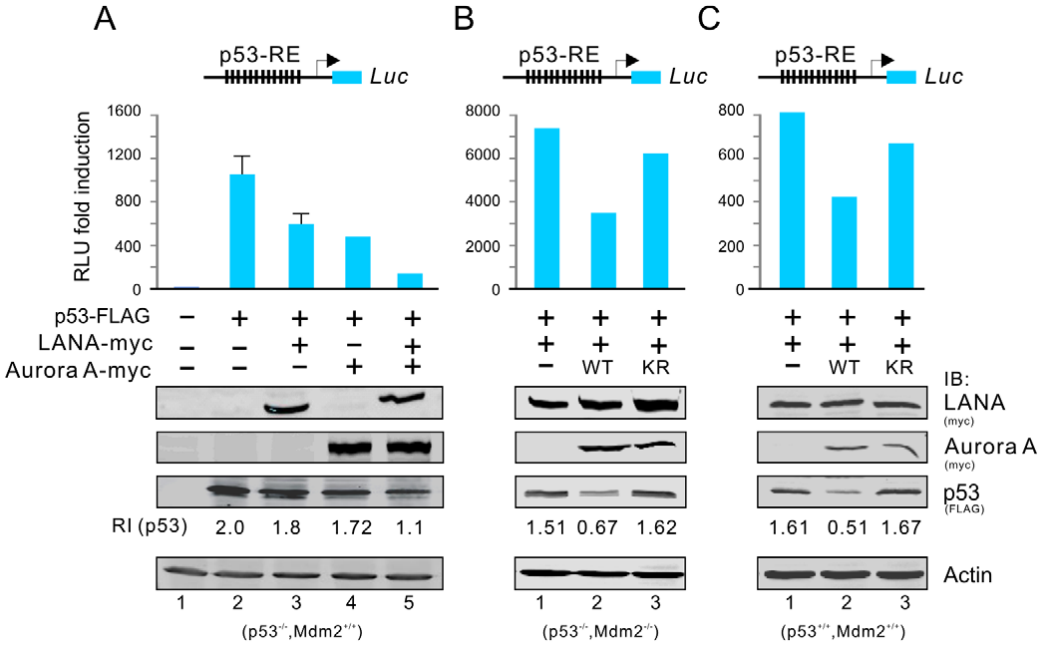
Coexpression of Aurora A enhances LANA-mediated repression of p53 transcriptional activity which depends on its kinase activity. Ten million of Saos-2 (A), MEF (B), or 293 (C) cells were co-transfected 3 µg p53 reporter plasmid with construct expressing LANA, Aurora A (WT or KR) or both. At 24 hr post-transfection, cells were harvested and lysed for luciferase assays. The expression levels of each target proteins were detected by westernblotting and shown at the bottom panels. β-actin blot was used as loading control.

To further determine if Aurora A is important for LANA to inhibit p53 function, we performed reporter assays in the p53-null Saos-2 cells by using a luciferase reporter driven by 13 consensus p53-binding sites. The results from reporter assays showed that both LANA and Aurora A individually reduced the transcriptional activity of p53 (Figure 4A, lane 3 and 4), and co-expression of Aurora A dramatically enhanced LANA-mediated inhibitory function of p53 expression (Figure 4A, lane 5). To determine if Aurora A-associated Mdm2 plays a role in cooperation of Aurora A with LANA as it relates to inhibition of p53, we also performed similar reporter assays in both *Mdm2* ^+/+^ and *Mdm2* ^−/−^ cells. The results further showed that in the absence or presence of Mdm2, Aurora A maintained its ability to collaborate with LANA in regards to inhibition of p53 transactivation (Figure 4B and C, lane 1 and 2). However, compared to wild type Aurora A, the kinase inactive mutant (KR) of Aurora A can greatly reverse LANA-mediated inhibition of p53 transactivation (Figure 4B and C, compare lane 2 with 3). This indicates that the KR mutant of Aurora A can act as a dominant negative molecule and that the kinase activity of Aurora A is required for LANA -induced repression of p53 transcriptional activity. Strikingly, in all three cell lines (*p53^−/−^*, *p53^−/−^Mdm2^−/−^* and *p53^+/+^Mdm2^+/+^*), we observed that co-expression of wild type Aurora A but not its KR mutant dramatically reduced the protein levels of p53 inhibited by LANA (Figure 4A, B and C, lower panels). Therefore, the kinase activity of Aurora A contributes to LANA-mediated degradation of p53 and so a reduction in p53 transcriptional activity.

### Aurora A enhances LANA^SOCS^-mediated ubiquitylation of p53 by inducing p53 phosphorylation

**Figure 5:**
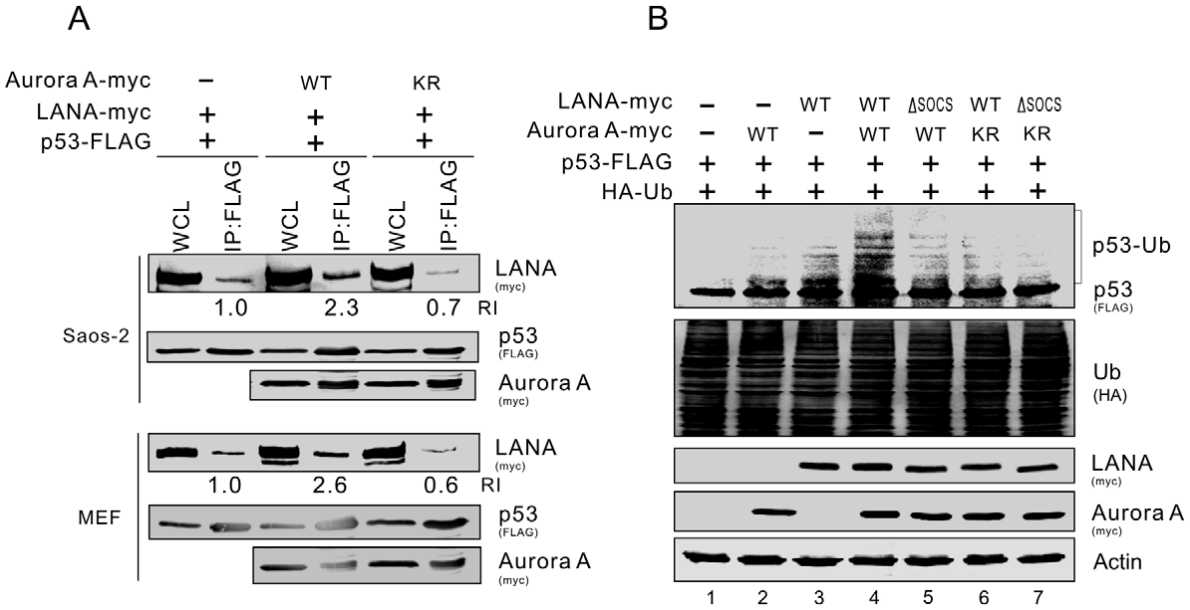
The active form of Aurora A kinase enhances LANA^SOCS^-mediated ubiquitylation of p53. (**A**) Active form of Aurora A kinase enhanced the interaction of p53 with LANA. Saos-2 or MEF cells were co-transfected p53-FLAG with pA3M-LANA plus either vector control, wild type (WT) Aurora A or its kinase inactive mutant (KR). At 36 hr post-transfection, the cells lysates were immunoprecipitated with anti-FLAG (M2) antibody, followed by immunoblotting with indicated antibodies. The relative intensity (RI) of p53-interacting LANA was presented by normalization with the protein amounts of both immunoprecipitated p53 and input LANA. (**B**) The LANA^SOCS^–mediated ubiquitylation of p53 was enhanced by Aurora A coexpression in a kinase activity-dependent manner. MEF cells were cotransfected with the plasmids expressing HA-Ub and p53-FLAG plus LANA-myc (WT or ΔSOCS) and Aurora A-myc (WT or KR). At 36 hr post-transfection with additional 6 hr MG132 treatment before harvest, cell lysates were analyzed by immunoblotting with as indicated antibodies.

Our previous work has shown that LANA can recruit the EC_5_S ubiquitin complex to degrade p53 [41]. To determine if Aurora A enhances LANA-mediated p53 proteolytic degradation, we assessed the stability of p53 in HEK293 cells transfected with LANA plus or minus Aurora A by exposure to cycloheximide for different time points. The results consistently showed lower levels of p53 in cells transfected with Aurora A than in cells transfected with a control vector (supplementary Figure S3). Treatment with cycloheximide for 210 min decreased p53 levels by almost 75% in Aurora A transfected cells compared to about 40% in the control cells (supplementary Figure S3, lane 4 and 8). Phosphorylation of p53 by Aurora A results in greater binding affinity with Mdm2 [40]. To determine if active Aurora A kinase enhanced the interaction of p53 with LANA, we monitored the association of p53 with LANA in the presence of wild type Aurora A or its inactive mutant KR by coimmunoprecipitation assays. Substantially more LANA protein was pulled down by p53 in the presence of wild type Aurora A than in the presence of the mutant control in both Saos-2 and MEF cell lines (Figure 5A).

To further determine if Aurora A-induced greater binding of p53 to LANA will result in LANA^SOCS^-mediated ubiquitylation of p53, we performed p53 ubiquitylation assays in MEF cells expressing different combinations of wild type LANA, mutants of SOCS-motif deleted mutant LANA (ΔSOCS), wild type Aurora A and its mutant KR. The results showed that there is substantially higher amount of ubiquitylated p53 in cells expressing wild type LANA and Aurora A than in cells expressing only wild type LANA or Aurora A (Figure 5B, compare lane 4 with 2 and 3). Consistently, mutation of the SOCS motif of LANA, the inactive form of Aurora A or both led to a dramatic reduction of ubiquitylated p53 (Figure 5B, compare lane 4 with 5, 6, and 7). This indicates that LANA^SOCS^-mediated ubiquitylation of p53 through the EC_5_S ubiquitin complex requires the kinase activity of Aurora A.

**Figure 6:**
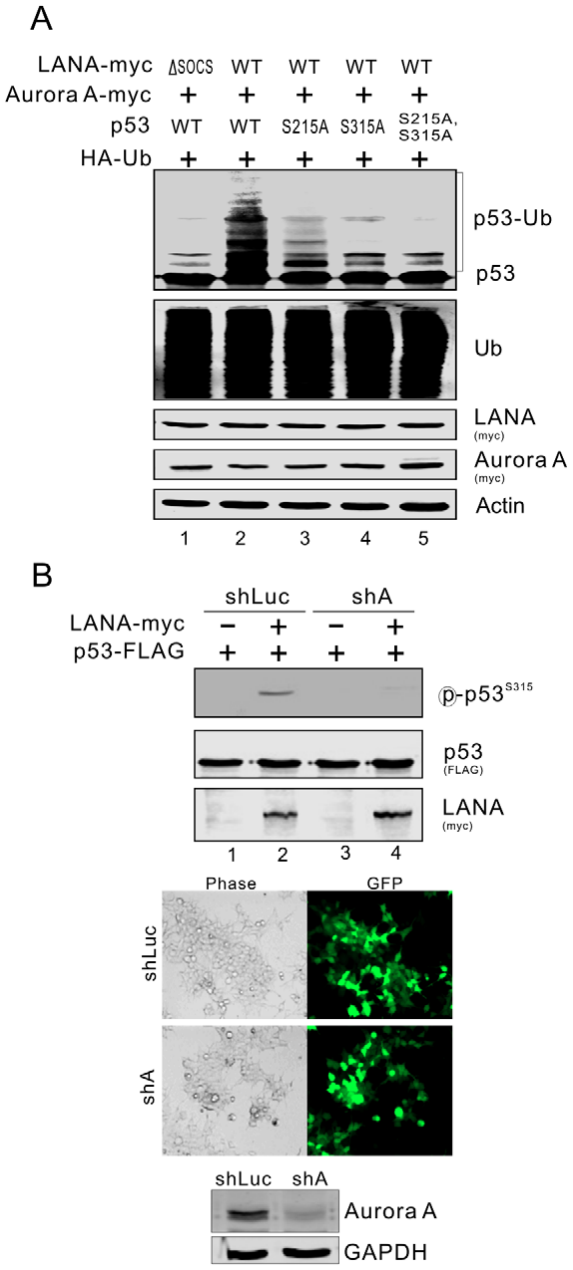
Aurora A-induced phosphorylation of p53 is a target for LANA^SOCS^-mediated ubiquitylation. (**A**) The Aurora A-induced phosphorylation on Ser 215 and Ser 315 was critical for LANA-mediated ubiquitylation of p53. MEF cells were cotransfected with different combinations of HA-Ub, HA-p53 (WT, S215A, S315A or S215A/S315A), LANA-myc (WT or ΔSOCS) and Aurora A-myc (WT or KR) as indicated in the figure. At 36 hr post-transfection with additional 6 hr MG132 treatment before harvest, cell lysates were analyzed by immunoblotting with as indicated antibodies. (**B**) LANA upregulated Aurora A-mediated phosphorylation of p53. MEF cells were cotransfected p53-FLAG with either plasmid expressing shAurora A or shLuc in the presence or absence of LANA-myc as indicated in the figure. At 36 hr post-transfection with additional 6 hr MG132 treatment before harvest, cell lysates were analyzed by immunoblotting with specific antibodies. The knockdown efficiency of shRNA against Aurora A is shown at the bottom panel.

Previous studies reported that Aurora A is able to phosphorylate p53 at Ser215 and Ser315 [36], [40]. To determine if LANA-mediated p53 ubiquitylation is dependent on Aurora A-induced phosphorylation of p53 on Ser215 or Ser315, we performed similar ubiquitylation assays by using wild-type, S215A or S315A variants of p53 with wild-type Aurora A and LANA in MEF cells. The results showed that compared with wild type p53, less ubiquitylated p53 appeared in cells expressing S215A, S315A or the S215A/S315A mutant of p53, and S315A had a greater impact than S215A (Figure 6A, lane 2 with 3, 4, and 5), suggesting that both Ser215 and Ser315 are phosphorylated by Aurora A and in turn facilitates LANA-induced ubiquitylation of p53. To further confirm that LANA does induce p53 phosphorylation through Aurora A, we coexpressed p53-FLAG with either LANA or empty vector in the presence of shAurora A or non-specific shLuc control in Saos-2 cells, followed by immunoblotting analysis with antibodies against the Ser315-phosphorylated p53. The results showed that LANA dramatically enhanced the level of phosphorylated p53 on Ser315 which dependent on Aurora A expression (Figure 6B, compare lane 2 with 1 and 3). Furthermore, Aurora A-mediated phosphorylation of p53 is required for LANA induced p53 ubiquitylation and degradation.

### Downmodulation of Aurora A attenuates LANA-mediated inhibition of p53 apoptotic activity

**Figure 7:**
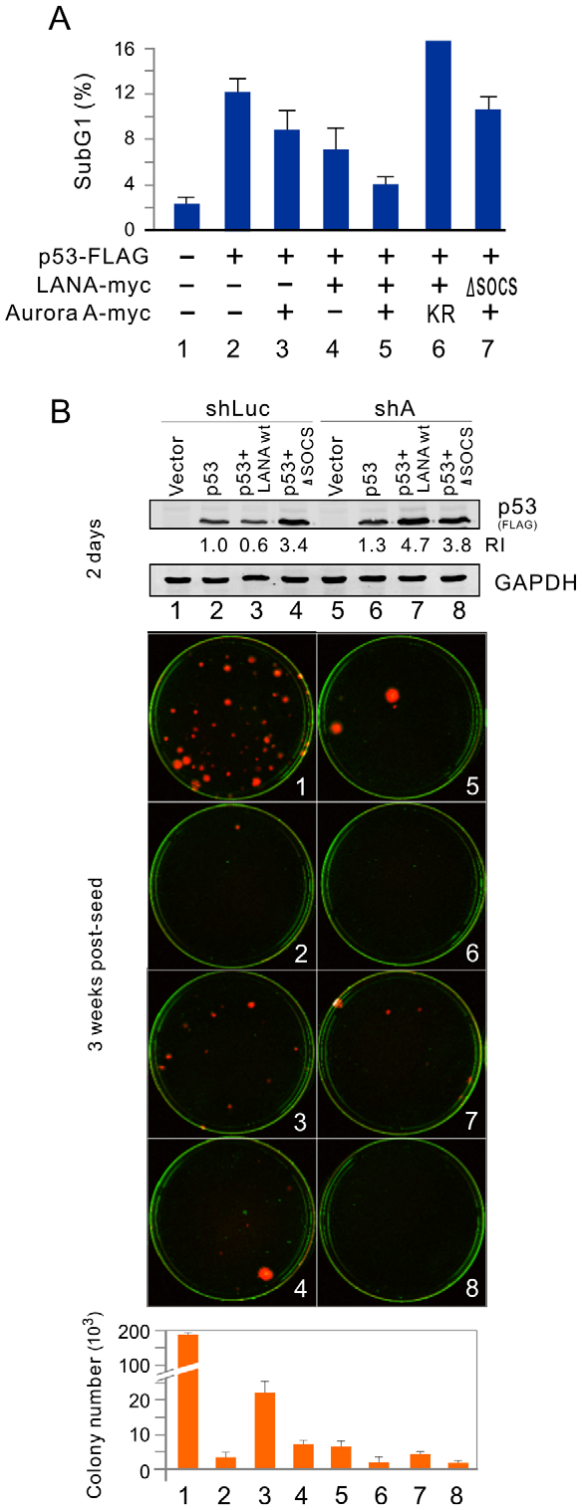
Aurora A is important for LANA to inhibit p53-induced apoptosis. (**A**) Saos-2 cells were cotransfected with different combination of plasmids expressing p53-FLAG, LANA-myc (wt or ΔSOCS), Aurora A-myc (wt or KR) or empty vectors as indicated in the figure. At 36 hr post-transfection, cell cycle profiles were analyzed with Flow cytometry. The percentage of subG1 population cells is presented. (**B**) Knockdown of Aurora A blocks LANA-mediated inhibition of p53 suppression function in colony formation. Saos-2 cells were cotransfected with different combination of plasmids expressing p53-FLAG, LANA-myc (wt or ΔSOCS) or empty vectors in the presence of shAurora A or shluc control as indicated in figure with number 1 to 8. *Upper panel*, Immunoblotting analysis of p53-FLAG expression at 48-hr posttransfection. *Middle panel*, a representative of colony formation after 3-week culture was shown. *Lower panel*, the data are presented as the average from two independent experiments.

It has been shown earlier that the increased expression of LANA can inhibit p53-induced apoptosis in *p53*-null Saos-2 cells [8]. To determine if Aurora A cooperation with LANA could affect p53-mediated apoptosis, Saos-2 cells transiently transfected with constructs expressing wild-type p53, plus either LANA, Aurora A or both LANA (WT) and Aurora A (WT or KR) in different combination, were subjected to cell cycle profile analysis. The results showed that the percentage of p53-induced apoptosis as determined by the subG1 population was greatly decreased with coexpression of LANA and Aurora A (Figure 7A, compare lane 2 with 3, 4 and 5, supplementary Figure S4). However, the combination of LANA with Aurora A mutant KR or LANA ΔSOCS with wild type Aurora A, resulted in a dramatic reversal of the levels of p53 mediated apoptosis (Figure 7A, compare lane 2 with 6 and 7, supplementary Figure S4). To further confirm that LANA does require Aurora A to inhibit p53-mediated apoptosis, we performed colony formation assays by coexpressing p53 with either wild type LANA or its deleted mutant (ΔSOCS), or empty vector in the presence or absence of Aurora A knockdown. Consistent with the expression level of p53 at 2 day posttransfection, the results of 3-weeks culture showed that p53 expression alone dramatically blocks colony formation, and wild type LANA but not its ΔSOCS mutant markedly reversed the inhibitory activities of p53 on colony formation (Figure 7B, compare lane 2 with 3 and 4). In contrast, there was no significant difference between LANA and p53 coexpression compared to p53 alone, once the expression of Aurora A was knocked down (Figure 7B, compare lane 6 with 7 and 8). Unexpectedly, we did observe that Aurora A knockdown alone remarkably reduced colony formation even without p53 coexpression (Figure 7B, compare lane 2 with 1). These suggest that Aurora A is important for LANA to inhibit p53-induced apoptosis by combining their phosphorylation and ubiquitylation activities.

### Aurora A knockdown enhances p53 stability and potently induces mitotic arrest and cell apoptosis in PEL cells

**Figure 8:**
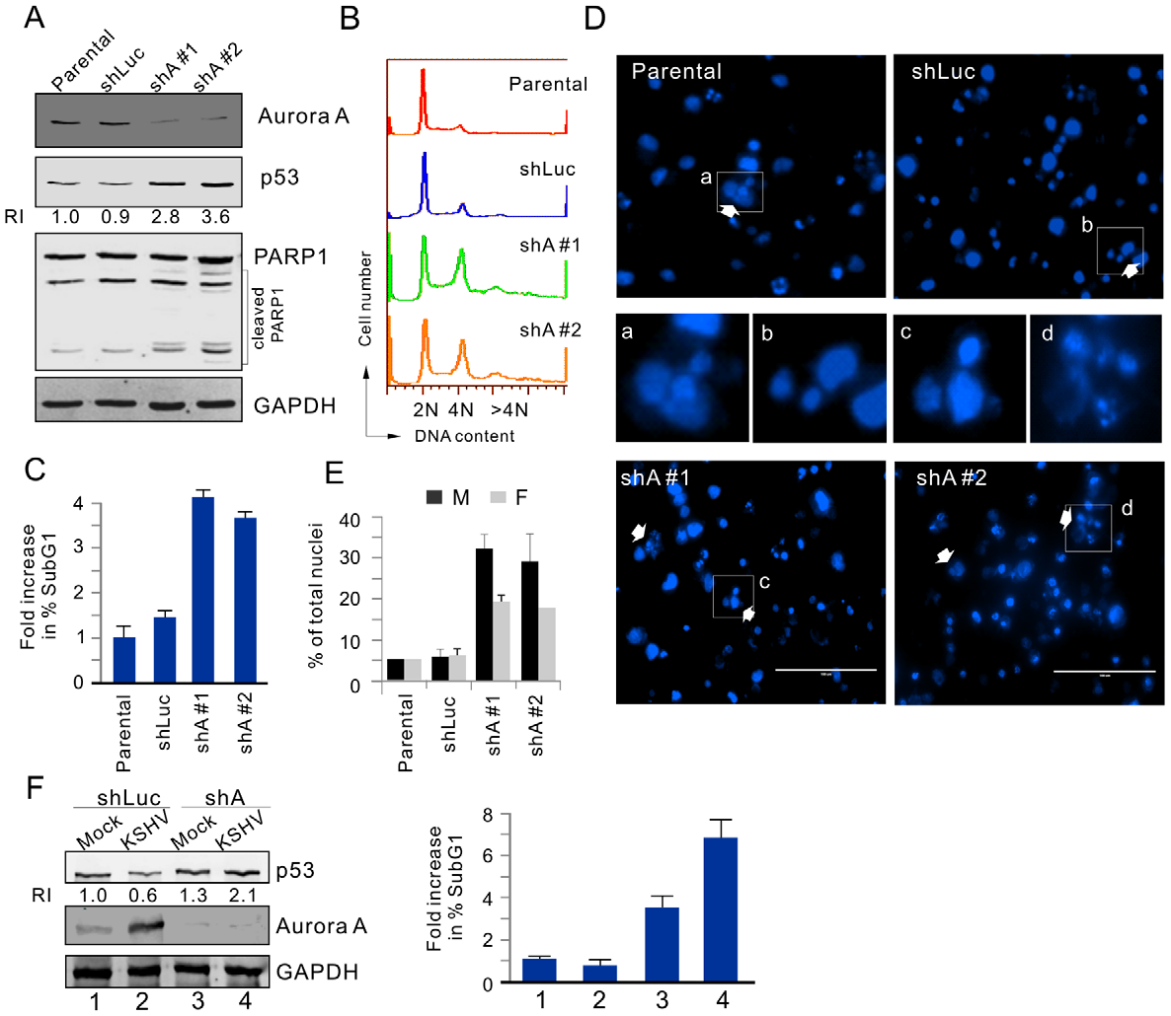
Downregulation of Aurora A induces cell cycle arrest and apoptosis in PEL cells. (**A**) Induction of PARP1 cleavage and increased p53 expression in cells with Aurora A knockdown. Parental BC3 cells and BC3 cells transduced by lentivirus expressing small hairpin against Aurora A (shA#1, shA#2) or luciferase (shLuc), were exposed to 0.1% sera for 18 hr. Cell lysates were subjected to immunoblotting as indicated antibodies in the figure. The relative instensity (RI) of p53 protein level is shown. (**B**) FACS analysis of BC3 cells with or without Aurora knockdown. Aurora A depletion induced cells with greater than 4N DNA content (Propidium iodide staining). (**C**) Fold changes in the subG1 population of cells with Aurora A knockdown, as analyzed by FACS. (**D**) DAPI staining of representative fields show multinucleation (white arrows), and inset fields show corresponding photographs to the white rectangles in the upper/lower panels. (**E**) Quantification of the multinucleated (M) and fragmented nuclei (F) in BC3 cells with or without Aurora A knockdown. (**F**) Aurora A knockdown dramatically increased p53 accumulation and subG1 population in KSHV-infected not uninfected cells. 293 (Mock) and 293-Bac36 (KSHV) cells were individually transduced by lentivirus expressing small hairpin against Aurora A (shA) and luciferase (shLuc) followed by immunoblotting and subG1 population analysis as described in panels A and C.

To further investigate the effect of Aurora A on the growth and survival of KSHV-infected B cells, the Aurora A expression in BC3 cells was knocked down by lentivirus-mediated shRNA against Aurora A. Lack of Aurora A expression rescued p53 expression about 3 fold in BC3 cells and greatly induced PARP1 cleavage, compared with BC3 cells transfected with control shRNA (Figure 8A). We also found that exposure of BC3 cells to low sera resulted in a marked increase of more than 4N DNA content in the cells, as well as the number of apoptotic cells due to inhibition of Aurora A expression (Figure 8B and C). Subsequently, the results of immunofluorescence analysis revealed that cells with shRNA against Aurora A exhibited a dramatic alteration in cellular and nuclear morphology. The cells were enlarged and exhibited an increased presence of multiple and fragmented nuclei (Figure 8D). Cells with multiple nuclei (>6N) could be readily seen. Furthermore, the fragmented nuclei were apparent in cells transduced with shAurora A. The proportion of multinucleated cells or apoptotic cells was progressively increased once Aurora A was knocked down (Figure 8D and E), indicating that inhibition of Aurora A in KSHV-infected cells was sufficient to induce cell apoptosis. In addition, the result of Aurora A knockdown led to dramatic increase of p53 accumulation and subG1 population in 293 cells with KSHV infection but not mock 293 cells (Figure 8F), further supporting the notion that Aurora A is targeted by KSHV for inhibition of p53-mediated apoptotic function.

## Discussion

**Figure 9:**
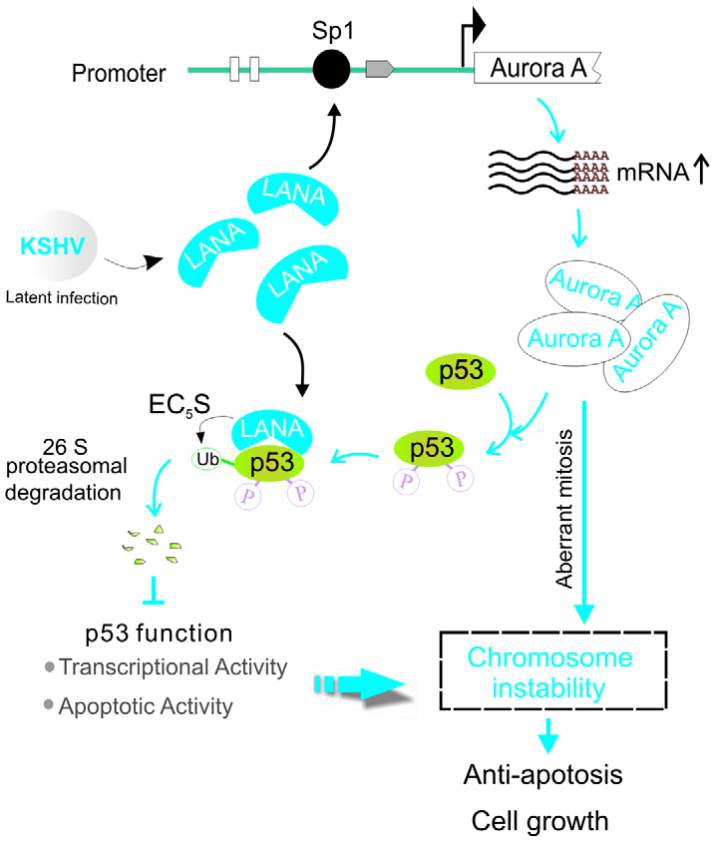
A model depicting the role of Aurora A targeted by LANA. In the KSHV latently infected cells, LANA upregulates Aurora A expression through targeting Sp1-binding site. The elevated Aurora A induced phosphorylation of p53 on Ser 215 and Ser315 which enhanced the binding affinity of p53 with LANA and promoted LANA^SOCS^-mediated ubiquitylation and degradation of p53, and thus inhibited p53 transcriptional and apoptotic activities. Loss of p53 function and aberrant expression of Aurora A trigger chromosome instability for cell survival and growth.

We and other groups have previously shown that LANA downregulates p53-associated pathways in the KSHV latently infected B lymphoma cells [8], [41]. In this study, we aimed to better understand the mechanism underlying LANA-mediated suppression of p53, a key protein already known to link with chromosomal instability and apoptosis. With this in mind, we performed apoptotic and chromosomal stability gene microarray analysis by using LANA stable expressing cell line and identified the Aurora A kinase, a mitotic checkpoint protein as one of the genes upregulated due to LANA expression. Further studies showed that expression of Aurora A upregulated by LANA is mainly through enhancing the binding capacity of transcriptional factor Sp1 to the Aurora A promoter. The elevated levels of Aurora A subsequently resulted in phosphorylation of p53 at Ser215 and Ser315 thus facilitating LANA-mediated ubiquitylation and destabilization of p53 (Figure 9). Therefore, LANA has a dual function in terms of regulating p53 by: 1) Enhancing Aurora A kinase expression to phosphorylate p53; and 2) Recruiting the EC_5_S ubiquitin complex to induce ubiquitylation of phosphorylated p53. A previous report showed that increased expression of Aurora A as a result of genetic mutations increased the growth and survival of HTLV-1-infected T cells [43]. Our data provide the first evidence showing that a viral protein can directly target the oncogenic Aurora A kinase for inhibition of p53 by enhancing the transcriptional activity of the Aurora A promoter.

The Aurora A gene is located at chromosome 20q13.2, a region frequently amplified and over-expressed in a variety of human tumors and cancer derived cell lines [27], [29], [44]. However, we did not observe a consistent difference in levels of Aurora A expression in KSHV-positive cell lines (BC3, BCBL1, JSC1 and BC1) and in KSHV negative cell lines (BJAB, Ramos, Loukes and DG75). To support our hypothesis that KSHV latent infection is directly associated with the elevated production of Aurora A, the levels of Aurora A transcripts in human primary cells with and without GFP-tagged KSHV infection were analyzed. Although the increased pattern of Aurora A (similar pattern for Aurora B, data not shown) transcripts wasn't consistently correlated with LANA expression within 7-days posttransfection, the base levels of Aurora A transcripts in KSHV-infected cells remained higher than that in KSHV-uninfected cells. One potential explanation for higher level of Aurora A mRNA transcripts by 2-days primary infection may be a benefit to rapidly establish KSHV latent infection. However, the fact that Aurora A transcript drops following persistent LANA expression after 2-days primary infection could be another benefit to the dynamical modulation of Aurora A by LANA and/or other factors during cell cycle progression. In agreement with the critical role of LANA on Aurora A expression, the evidence have showed that increased transient expression of LANA can upregulate Aurora A expression in a dose-dependent manner, and that knock-down of LANA in KSHV-positive cell lines decreased both the transcript and protein levels of Aurora A. In addition, immunohistochemistry assays further revealed that expression of high levels of Aurora A in cells from KS tissue is correlated with the presence of LANA in KSHV-positive cells.

There are at least 20 phosphorylation sites reported for human p53 [45]. Most of the amino terminal specific phosphorylation sites prevents Mdm2-mediated ubiquitylation and thus stabilizes p53 [45]. In contrast, phosphorylation of p53 at its carboxyl terminus often promotes p53 degradation [45]. For example, phosphorylation of Ser362/366 by NF-κB induces p53 degradation [46]. In regards to the effect of Aurora A-induced phosphorylation of p53, Aurora A is able to induce Mdm2-mediated p53 degradation by phosphorylating Ser315 [40], and abrogate the DNA binding and transactivation activity of p53 by phosphorylating Ser215 not Ser315 [36]. Although this is somehow controversial, these reports implicate that p53 is a physiological substrate of Aurora A and that Aurora A may exert its regulatory functions on p53 through phosphorylation of Ser215 and Ser315 in different cell types. Our data showed that mutation of p53 at Ser315 dramatically reduced LANA-mediated ubiquitylation of p53 in the presence of Aurora A. However, mutation at Ser215 also reversed the inhibition of p53 ubiquitylation induced by LANA. Therefore, the virus has broad control of the kinase activity of Aurora A in its inhibition of p53 which overcomes the issue of cell type specificity. In addition, identification of Aurora A kinase for Ser315 phosphorylation does not rule out possible involvement of other cell cyclin-dependent kinases, as precedents for multiple distinct kinases targeting the same phosphorylation site of p53 (ATM and ATR for Ser15) have been reported [47], [48].

To determine the role of LANA in destabilization of p53 phosphorylated by Aurora A kinase, we compared the effect of wild-type LANA and mutant LANA (ΔSOCS) lacking the ability to ubiquitinate p53 and showed that the ubiquitination activity of LANA is critical for destabilization of p53 after induction of Aurora A expression. This suggests that Aurora A phosphorylates p53, thus enhancing LANA-mediated degradation of p53. Recent reports show that KSHV targets Mdm2 to deregulate the p53 tumor suppressor pathway [49], [50]. Interestingly, our reporter assay found that the protein level and transactivation activity of p53 were dramatically reduced by LANA together with Aurora A in an Mdm2-independent manner. Phosphorylation of p53 by Aurora A results in greater binding affinity to Mdm2 and acceleration of its degradation [40]. However, the possibility exists that Mdm2 contributes to ubiquitylation and degradation of Aurora A-mediated phosphorylation of p53 in LANA-expressing cells. Moreover, wild type Aurora A but not its kinase deficient mutant enhanced interaction of p53 with LANA, and suggests that Aurora A-induced phosphorylated p53 potentially has a higher affinity for LANA than the native p53. However, whether Aurora A is directly involved in a complex which contains p53 and LANA to increase their ability to interact remains to be further investigated.

Aurora is a subfamily of serine/threonine protein kinases that plays critical roles in centrosome cycling, spindle assemble, chromosome segregation and cell division [51], [52]. The observation of growth arrest of cells at the G2-M phase after silencing of Aurora A kinase in KSHV positive PEL cells, suggests that degradation of p53 phosphorylated at Ser215 and Ser315, has physiological relevance in allowing progression of normal cell proliferation cycle. In regard to our observation that Aurora A knockdown alone also induces less colony formation in the *p53*-null cells, the function of Aurora A on p53 is likely to be the major but not unique signaling pathway for blocking apoptosis. Our data also indicated that coexpression of LANA and Aurora A was able to inhibit p53-mediated apoptosis in the *p53*-null cell line, Saos-2. Based on our ubiquitylation assays and protein stability assays, Aurora A is important for LANA to suppress p53-dependent apoptosis by phosphorylating p53, followed by its ubiquitination and degradation. This function of LANA may explain in part why KSHV latent infection causes oncogenic transformation in mammalian cells [53].

In summary, Aurora A is a candidate target for inhibition of tumor growth in a broad range of cancers including pancreatic and leukemia cells in which it is amplified or induced. Our studies demonstrates that transcription activation of Aurora A by the viral protein LANA leads to induced expression of this protein in KSHV infected cells. Down-regulation of Aurora A by RNA interference induces cell-cycle arrest, aberrant chromosomal segregation and apoptosis (about 4-fold higher) in PEL cells, suggesting that Aurora A could be a promising therapeutic target in PEL cells.

## Materials and Methods

### Ethics statement

De-indentified Human peripheral blood mononuclear cells (PBMCs) were obtained from the University of Pennsylvania CFAR Immunology Core. The Core maintains an IRB approved protocol in which Declaration of Helsinki protocols were followed and each donor gave written, informed consent.

### Plasmids, antibodies and cell lines

DNA constructs expressing LANA full length and SOCS-box mutant in the pA3M vector were described previously [22], [41]. The p53 reporter plasmid containing 13 copies of p53-binding sites at upstream of the luciferase gene was provided by Wafik S. EI-Deiry (Milton Hersley Medical School, Hersley) [54]. pcDNA4(TO/myc-His)-Aurora A wild type and kinase inactive K/R mutant were provided by Erich A. Nigg (Max-Planck Institute of Biochemistry, Martinsried, Germany). HA-p53 WT, S215A, S315A and S215A/S315A mutant were a kind gift from Jin Q. Cheng (University of South Florida, Tampa, Florida). The Aurora A promoter-driven luciferase plasmids pGL3-pAurora A (E2F+Sp1+Ets) were generated by PCR amplicon inserted into pGL3 vector with *Kpn*I and *Bgl*II digestion. The deleted (ΔE2F, ΔEts, ΔSp1 or ΔSp1+Ets) mutants of Aurora A promoter are derived from pGL3-pAurora A. The monoclonal antibody anti-myc (9E10) and HA (12CA5) were prepared from hybridoma cultures. Goat ployclonal antibody against Aurora A (Ark-1, N-20) was purchased from Santa Cruz Biotechnology, Inc. (Santa Cruz, CA). Mouse monoclonal antibody against FLAG epitope (M2) was purchased from Sigma-Aldrich Corp. (St. Louis, MO). Rabbit antibody against phosphorylated p53 (Ser315) was purchased from Cell Signaling.

The B lymphoma cell lines BC3 (KSHV positive) and DG75 (KSHV negative) were cultured in RPMI 1640 medium supplemented with 7% fetal bovine serum, 2 mM L-glutamine, and penicillin-streptomycin (5 U/ml and 5 µg/ml, respectively). HEK 293, MEF, and Saos-2 cells were cultured in Dulbecco's modified Eagle's medium (DMEM) supplemented with 5% fetal bovine serum, 2 mM L-glutamine, and penicillin-streptomycin (5 U/ml and 5 µg/ml, respectively). All cell lines were grown at 37°C in a humidified environment supplemented with 5% CO_2_.

### Immunohistochemistry

Slides mounted with sections of paraffin-embedded, archival, deidentified KS tissue specimens were a generous gift from Michael Feldman (Department of Pathology and Laboratory Medicine, Hospital of the University of Pennsylvania, Philadelphia, PA). Slides were deparaynized in xylene and rehydrated through graded alcohols (70, 80, and 100% alcohol; 5 min each). Endogenous peroxidase activity was blocked with 3% hydrogen peroxide for 10 min. Following antigen retrieval in 10 mM sodium citrate buffer (pH 6.0), samples were blocked with 10% normal rabbit/goat serum prior to incubation with primary antibodies overnight at 4°C. Secondary antibody (biotinylated anti-rabbit/goat IgG) and streptavidin–peroxidase conjugate (S-P kit, DAKO) were added according to the manufacturer's instructions. Color reaction was developed using diaminobenzidine chromogen solution (Liquid DAB, DAKO).

### KSHV virion purification and primary infection

The 293/Bac36 (GFP-KSHV) cells were subjected to induction by TPA and Sodium butyrate for KSHV virion production. After induction, the supernatant of culture medium was collected and filtered through 0.45 µm filter, and viral particles were spun down at 25,000 rpm for 2 h, at 4°C. The concentrated virus was collected and used for infection or viral DNA quantitation. For primary infection, 10×10^6^ PBMC cells were incubated with virus suspension in 1 ml of RPMI 1640 (10% FBS) medium in the presence of 5 µg/ml Polybrene (Sigma, Marlborough, MA) and incubated for 4 hrs at 37°C. Cells were centrifuged for 5 min at 1500 rpm, the supernatant discarded, pelleted cells were resuspended in fresh RPMI 1640 (10% FBS) medium in 6 well plates and cultured at 5% CO_2_, 37°C humidified incubator. The positive infection was checked by detection of LANA expression.

### Quantitative real-time PCR

Total RNA from cells was extracted using Trizol reagent and cDNA was made with a Superscript II reverse transcription kit (Invitrogen, Inc., Carlsbad, CA). The primers for real-time PCR were as followings: for Aurora A: 5′-GGAGAGCTTAAAATTGCAGATTTTG-3′ and 5′-GGCAAACACATACCAAGAGA CCT-3′; for LANA: 5′-CATACGAACTCCAGGTCTGTG-3′ and 5′-GGTGGAAGAGCC CATAATCT-3′; for vCyclinD: 5′-TAATAGAGGCGGGCAATGAG-3′ and 5′-ACTCCTTT TCCCGCCAAGAAC-3′; for vFLIP: 5′-GTGTAAGAATGTCTGTGGTGTGC-3′ and 5′- GCGGAATGTCTGTTTCGTGC-3′; and for GAPDH: 5′-CTCCTCTGACTTCAACAGC G-3′ and 5′-GC CAAATTCGTTGTCATACCAG-3′. The cDNA was amplified by using 10 µl of Master Mix from the DyNAmo SYBR green quantitative real-time PCR kit (MJ Research, Inc.), 1 µM of each primer, and 2 µl of the cDNA product in a 20-µl total volume. Thirty cycles of 1 min at 94°C, 30 s at 55°C, and 40 s at 72°C were followed by 10 min at 72°C in an MJ Research Opticon II thermocycler (MJ Research, Inc., Waltham, MA). A melting curve analysis was performed to verify the specificities of the amplified products. The values for the relative levels of change were calculated by the “delta delta threshold cycle” (ΔΔC_T_) method and each sample were tested in triplicates.

### RNA interference

Small hairpin RNAs (shRNA) complementary to the C-terminal (GCTAGGCCACAACACATCT) fragment of LANA as described previously [41], or N-terminal (GTCTTGTGTCCTTCAAATT) fragment of Aurora A was individually cloned into pGIPz vector according to the manufacture's instructions (Open Biosystem, Inc, Huntsville, AL) to generate shLANA and shAurora A constructs. pGIPz vector with luciferase target sequence (shLuc) was used as a control. Fifteen million BC3 cells were transfected by electroporation (220v, 975 µF, 0.4-cm-gap cuvette) with 20 µg of shLANA (or shLuc). BC3 knockdown stable cells were selected and maintained in 4 µg/ml puromycin.

### Immunoprecipitation and immunoblotting

Transfected cells were harvested, washed with ice-cold PBS, and lysed in ice-cold RIPA buffer. Cell debris was removed by centrifugation at 21,000× g (10 min and 4°C), and the supernatant was transferred to a fresh microcentrifuge tube. Lysates were then precleared by end-over-end rotation with normal mouse serum and a 1∶1 mixture of Protein A and Protein G-conjugated Sepharose beads (1 hr, 4°C). Beads were spun out, and the supernatant was transferred to a fresh microcentrifuge tube. The protein of interest was captured by rotating the remaining lysate with 1 µg of appropriate antibody overnight at 4°C. Immune complexes were captured with 30 µl of a 1∶1 mixture of Protein A and Protein G Sepharose beads, pelleted, and washed five times with ice-cold RIPA buffer. For immunoblotting assays, input lysates and immunoprecipitated (IP) complexes were boiled in Laemmli buffer, fractionated by SDS-PAGE, and transferred to a 0.45-µm nitrocellulose membrane. The membrane was then probed with appropriate antibodies followed by incubation with appropriate infrared-tagged secondary antibodies and finally was scanned with an Odyssey Infrared scanner (Li-Cor Biosciences, Lincoln, NE). Densitometric analysis was performed with the Odyssey scanning software.

### Reporter assays

Reporter assay was essentially performed as described previously [55]. Briefly, the cells were transiently transfected with the combined plasmids as indicated. The differences in the amounts of total DNA were normalized with vector control to reach the same amount of total transfected DNA. At 24 h post-transfection, cells were harvested and lysed in reporter lysis buffer (Promega Inc., Madison, WI). A 40 µl aliquot of the lysate was transferred to a 96-well plate. Luciferase activity was measured using an LMaxII384 luminometer (Molecular Devices, Sunnyvale, CA) by automatically injecting 25 µl of luciferase substrate into each well and integrating the luminescence for 20 s post-injection. The results represent experiments performed in duplicate.

### Chromatin immunoprecipitation assay

The chromatin immunoprecipitation (ChIP) experiments were done essentially as previously described with some modifications. BC3 cells (10×10^6^) with LANA or control knockdown were cross-linked with 1.1% (v/v) formaldehyde, 100 mM NaCl, 0.5 mM EGTA, and 50 mM Tris-HCl (pH 8.0) in growth medium at 37°C for 10 min, then at 4°C for 50 min. Formaldehyde was quenched by adding 0.05 vol 2.5 M glycine. Fixed cells were washed with PBS, incubated for 15 min in 15 ml of 10 mM Tris-HCl (pH 8.0), 10 mM EDTA, 0.5 mM EGTA, and 0.25% (v/v) Triton X-100, followed by 15 min in 15 ml of 10 mM Tris-HCl (pH 8.0), 1 mM EDTA, 0.5 mM EGTA, and 200 mM NaCl, and finally sonicated in 1 ml of 10 mM Tris-HCl (pH 8.0), 1 mM EDTA, 0.5 mM EGTA, 1% (w/v) SDS plus 1 mM PMSF, 1 µg/ml aprotonin, leupettin, and pestatin to an average fragment size of 300–500 bp. 20% of solubilized chromatin extracts were saved for input followed with cross-link reverse step, and the remaining were clarified by centrifugation at 12,000 g, and diluted to 6 OD_260_ U/ml in IP buffer [140 mM NaCl, 1% (w/v) Triton X-100, 0.1% (w/v) sodium deoxycholate, 1 mM PMSF, 100 µg/ml salmon sperm DNA, and 100 µg/ml BSA]; preincubated for 1 h at 4°C with 10 µl/ml 50% (v/v) Protein A-agarose (Invitrogen Life Technologies, Camarillo, CA) with normal mouse/rabbit sera; reconstituted in PBS, and washed several times in IP buffer. Aliquots (600 µl) were incubated with 20 µg of each specific antibody for overnight at 4°C. Immune complexes were separated into bound and unbound complexes with protein A-agarose and cross-links were reversed by treatment at 65°C overnight. After treatment with RNase A and proteinase K, samples were extracted once with phenol/chloroform, and the DNA was precipitated with 2 volumes of ethanol. Precipitated DNA was pelleted, washed once with 70% ethanol, dried, and resuspended in 100 µl of water. The DNA was analyzed by quantitative PCR with Aurora promoter primers (sense: 5′- TTCGATCGACCAGCTGGTCC GGTTCT -3′, anti-sense: 5′- TTCTCGAGCACTTGCTCCCTAAGAAC -3′).

### In vivo ubiquitylation assay

MEF cells (15×10^6^) were transfected by electroporation with appropriate combination of plasmids expressing HA-Ub (5 µg), p53-FLAG (or HA-p53) (8 µg), Aurora A-myc (8 µg) and LANA-myc (8 µg). Cells were incubated for 36 hr and pretreated for an additional 6 hr with 20 µM MG132 (Biomol International) before harvesting. The extent of ubiquitylation of p53 proteins was determined by western blot analysis using the FLAG-specific antibody (M2) or p53 (Do-1) antibody.

### Colony formation assay

Ten million of Saos-2 cells were typically transfected using electroporation with different combinations of expression plasmids as shown in the text. At 48 hr posttransfection, 1×10^4^ transfected cells were cultured in the selection medium (DMEM supplemented with 2 µg/ml puromycin). After 14 days, cells were fixed on the plates with formaldehyde and stained with 0.1% crystal violet. The amount of the colonies in each dish was scanned by Li-Cor Odyseesy and counted.

### Cell cycle assay

Cells were harvested and washed with ice-cold PBS and fixed in 70% cold ethanol overnight at 4°C. The fixed cells were then stained with PBS containing 40 µg/ml of propidium iodide (Sigma, St Louis, MO), 200 µg/ml of RNase A (Sigma) and 0.05% Triton X-100 for 1 h at 4°C in dark. Different cell cycle phases cells were characterized by using a FACSCalibur (BD Biosciences, San Joe, CA) and the results were analyzed using the FlowJo software (Tree star, Ashland, OR). For cytometric assessment of Aurora A expression profile at G1, S and G2 phase, 10×10^6^ transfected cells were fixed by 3% paraformaldelhyde for 20 min at room temperature, and washed with PBS and subsequently blocked in 1% BSA with 0.1% Triton X-100, followed by incubation with 1 µg goat anti-Aurora A antibody (Ark-1, N-20) or normal IgG serum for overnight at at 4°C. Cells were washed three times with blocking buffer and exposed to secondary chicken anti-goat antibody (1∶500 dilution) conjugated with Alexa Fluor 488 for 1 hr at room temperature, followed by three washes with PBS, and staining with propidium iodide and RNase A for cell cycle.

## Supporting Information

Figure S1 Quantitative analysis of vCyclinD(ORF72), vFLIP(K13) and LANA(ORF73). Total RNAs from BC3 cells with transiently LANA knockdown (shLANA) or control firefly luciferase (shLuc) knockdown were prepared and individually transcribed to cDNA. The qRT-PCR analysis with the primers for ORF72, K13 and ORF73 were performed using the Power SYBR green PCR Master Mix with GAPDH as a control. The relative quantitation (RQ) of corresponding vCyclinD, vFLIP and LANA mRNAs was individually presented by dark, light and blank rectangles. Error bars indicate standard deviations from three separate experiments. * *p*>0.05; **p<0.01.
(TIF)

Figure S2 LANA enhanced the transcriptional level of Aurora A promoter. (**A**) The schematic representation of the Aurora A gene promoter-driven luciferase. The putative binding sites for transcriptional factors E2F, Sp1 and Ets are indicated. The relative position indicates the distance from the major transcriptional initiation site. (**B**) HEK293 or DG75 cells cotransfected pGL3-basic or pGL3-Aurora A with either pA3M-LANA or empty vector, were harvested at 24 h post-transfection. The cell lysate were subjected to luciferase reporter assay. The results were presented by the RLU (relative luciferase unit) fold compared to pGL3-basic with vector alone. Data is presented as means±SD of three independent experiments. The immunoblotting (IB) results of myc-tagged LANA and GAPDH were shown at the bottom panels. (**C**) Reporter assay of pGL3- Aurora A promoter co-transfection with increasing amount (0, 5, 10, 15, 20 µg) of pA3M-LANA. At 24 h post-transfection, cells were harvested and subjected to luciferase reporter assay as described in panel B. The results were presented by the RLU fold compared to pGL3-Aurora A with empty vector alone.
(TIF)

Figure S3 Coexpression of Aurora A enhanced LANA-mediated p53 instability. HEK 293 cells were co-transfected pA3M LANA with Aurora A. At 36 h posttransfection, cells were treated with 40 µg/ml cycloheximide (CHX) for different time points. Then cells were harvested and lysed for western blot analysis. The amount of p53 was quantified by band density and is shown relative to the amount of p53 expressed in the vector-transfected cells.
(TIF)

Figure S4 Representative data sets showing cell cycle profiles of Saos-2 cells cotransfected with different combination of plasmids expressing p53-FLAG, LANA-myc (WT or ΔSOCS) or Aurora A-myc (WT or KR) as indicated in the Figure 7A (lanes 1 to 7).
(TIF)
