# Supplementary figures and images for: Kaposi's Sarcoma Herpesvirus Upregulates Aurora A Expression to Promote p53 Phosphorylation and Ubiquitylation
Source: PLoS Pathog. 2012 Mar 1;8(3):e1002566. doi: 10.1371/journal.ppat.1002566 (PMC3291660; doi:10.1371/journal.ppat.1002566)

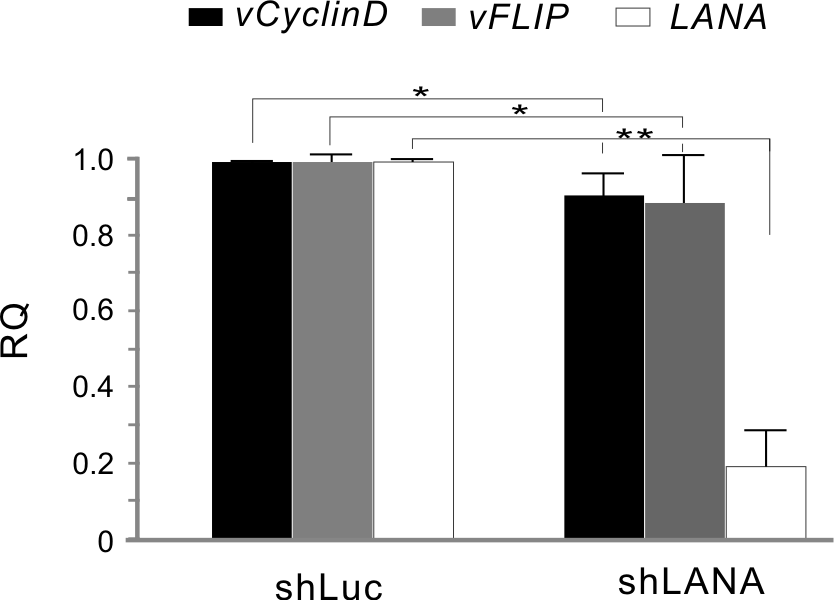

Supplement: Figure S1 — Quantitative analysis of vCyclinD(ORF72), vFLIP(K13) and LANA(ORF73). Total RNAs from BC3 cells with transiently LANA knockdown (shLANA) or control firefly luciferase (shLuc) knockdown were prepared and individually transcribed to cDNA. The qRT-PCR analysis with the primers for ORF72, K13 and ORF73 were performed using the Power SYBR green PCR Master Mix with GAPDH as a control. The relative quantitation (RQ) of corresponding vCyclinD, vFLIP and LANA mRNAs was individually presented by dark, light and blank rectangles. Error bars indicate standard deviations from three separate experiments. * p>0.05; **p<0.01. (TIF) [file ppat.1002566.s001.tif]

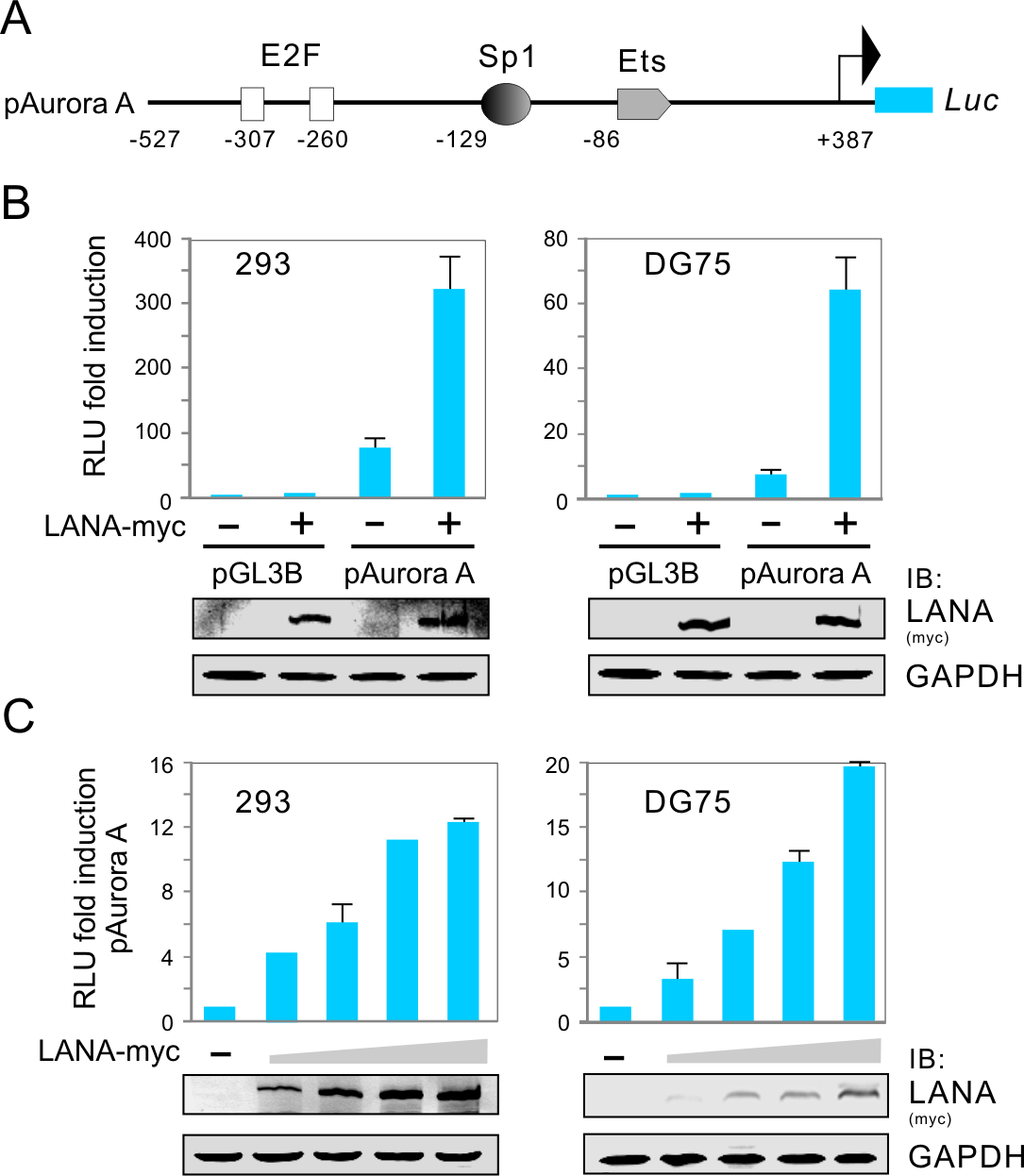

Supplement: Figure S2 — LANA enhanced the transcriptional level of Aurora A promoter. (A) The schematic representation of the Aurora A gene promoter-driven luciferase. The putative binding sites for transcriptional factors E2F, Sp1 and Ets are indicated. The relative position indicates the distance from the major transcriptional initiation site. (B) HEK293 or DG75 cells cotransfected pGL3-basic or pGL3-Aurora A with either pA3M-LANA or empty vector, were harvested at 24 h post-transfection. The cell lysate were subjected to luciferase reporter assay. The results were presented by the RLU (relative luciferase unit) fold compared to pGL3-basic with vector alone. Data is presented as means±SD of three independent experiments. The immunoblotting (IB) results of myc-tagged LANA and GAPDH were shown at the bottom panels. (C) Reporter assay of pGL3- Aurora A promoter co-transfection with increasing amount (0, 5, 10, 15, 20 µg) of pA3M-LANA. At 24 h post-transfection, cells were harvested and subjected to luciferase reporter assay as described in panel B. The results were presented by the RLU fold compared to pGL3-Aurora A with empty vector alone. (TIF) [file ppat.1002566.s002.tif]

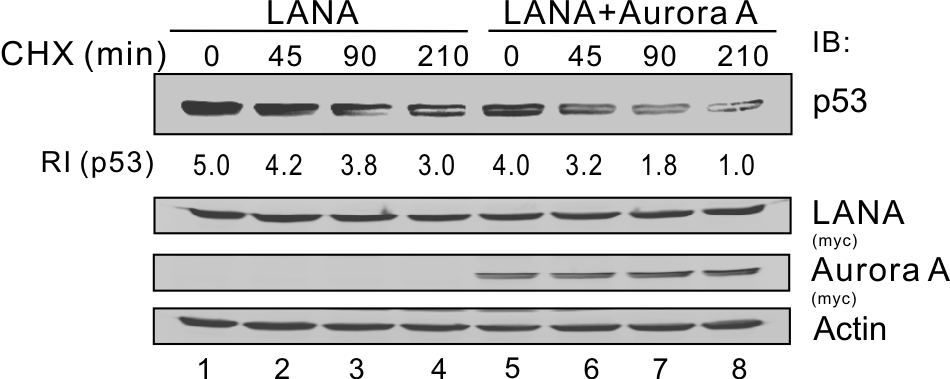

Supplement: Figure S3 — Coexpression of Aurora A enhanced LANA-mediated p53 instability. HEK 293 cells were co-transfected pA3M LANA with Aurora A. At 36 h posttransfection, cells were treated with 40 µg/ml cycloheximide (CHX) for different time points. Then cells were harvested and lysed for western blot analysis. The amount of p53 was quantified by band density and is shown relative to the amount of p53 expressed in the vector-transfected cells. (TIF) [file ppat.1002566.s003.tif]

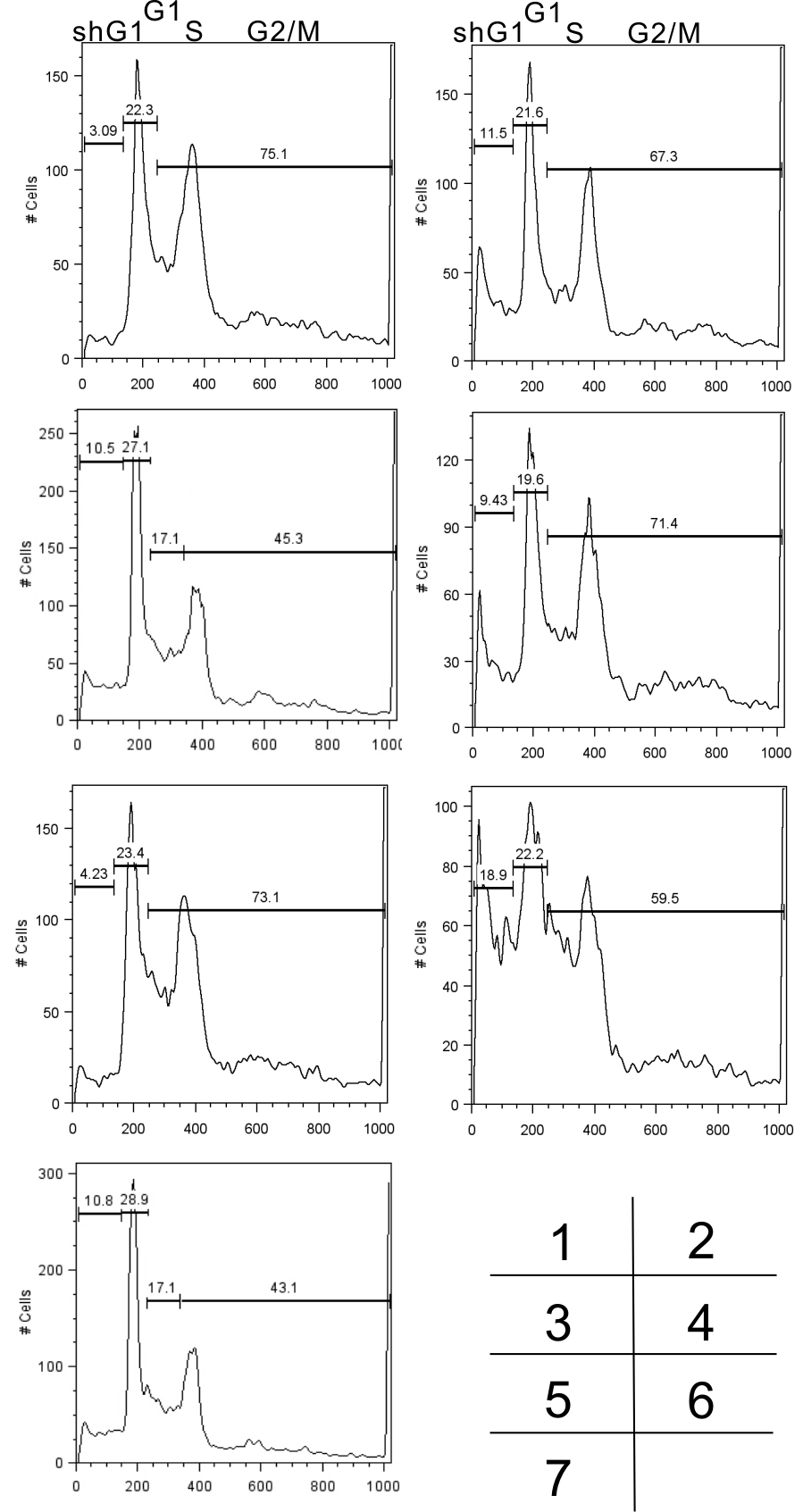

Supplement: Figure S4 — Representative data sets showing cell cycle profiles of Saos-2 cells cotransfected with different combination of plasmids expressing p53-FLAG, LANA-myc (WT or ΔSOCS) or Aurora A-myc (WT or KR) as indicated in the Figure 7A (lanes 1 to 7). (TIF) [file ppat.1002566.s004.tif]
